# Association of BCG Vaccination in Childhood With Subsequent Cancer Diagnoses: A 60-Year Follow-up of a Clinical Trial

**DOI:** 10.1001/jamanetworkopen.2019.12014

**Published:** 2019-09-25

**Authors:** Nicholas T. Usher, Suyoung Chang, Robin S. Howard, Adriana Martinez, Lee H. Harrison, Mathuram Santosham, Naomi E. Aronson

**Affiliations:** 1 Infectious Diseases Division, Uniformed Services University of the Health Sciences, Bethesda, Maryland; 2 College of Agriculture and Life Sciences, Cornell University, Ithaca, New York; 3 Division of Vaccines and Related Product Applications, US Food and Drug Administration, Silver Spring, Maryland; 4 Department of Research Programs, Walter Reed National Military Medical Center, Bethesda, Maryland; 5 Infectious Diseases Epidemiology Research Unit, University of Pittsburgh, Pittsburgh, Pennsylvania; 6 Health Systems Program, Bloomberg School of Public Health, Johns Hopkins University, Baltimore, Maryland; 7 Center for American Indian Health, Bloomberg School of Public Health, Johns Hopkins University, Baltimore, Maryland

## Abstract

**Importance:**

The BCG vaccine is currently the only approved tuberculosis vaccine and is widely administered worldwide, usually during infancy. Previous studies found increased rates of lymphoma and leukemia in BCG-vaccinated populations.

**Objective:**

To determine whether BCG vaccination was associated with cancer rates in a secondary analysis of a BCG vaccine trial.

**Design, Setting, and Participants:**

Retrospective review (60-year follow-up) of a clinical trial in which participants were assigned to the vaccine group by systematic stratification by school district, age, and sex, then randomized by alternation. The original study was conducted at 9 sites in 5 US states between December 1935 and December 1998. Participants were 2963 American Indian and Alaska Native schoolchildren younger than 20 years with no evidence of previous tuberculosis infection. Statistical analysis was conducted between August 2018 and July 2019.

**Interventions:**

Single intradermal injection of either BCG vaccine or saline placebo.

**Main Outcomes and Measures:**

The primary outcome was diagnosis of cancer after BCG vaccination. Data on participant interval health and risk factors, including smoking, tuberculosis infection, isoniazid use, and other basic demographic information, were also collected.

**Results:**

A total of 2963 participants, including 1540 in the BCG vaccine group and 1423 in the placebo group, remained after exclusions. Vaccination occurred at a median (interquartile range) age of 8 (5-11) years; 805 participants (52%) in the BCG group and 710 (50%) in the placebo group were female. At the time of follow-up, 97 participants (7%) in the placebo group and 106 participants (7%) in the BCG vaccine group could not be located; total mortality was 633 participants (44%) in the placebo group and 632 participants (41%) in the BCG group. The overall rate of cancer diagnosis was not significantly different in BCG vaccine vs placebo recipients (hazard ratio, 0.82; 95% CI, 0.66-1.02), including for lymphoma and leukemia. The rate of lung cancer was significantly lower in BCG vs placebo recipients (18.2 vs 45.4 cases per 100 000 person-years; hazard ratio, 0.38; 95% CI, 0.20-0.74; *P* = .005), controlling for sex, region, alcohol overuse, smoking, and tuberculosis.

**Conclusions and Relevance:**

Childhood BCG vaccination was associated with a lower risk of lung cancer development in American Indian and Alaska Native populations. This finding has potentially important health implications given the high mortality rate associated with lung cancer and the availability of low-cost BCG vaccines.

## Key Points

**Question:**

What is the association of BCG vaccination during childhood with subsequent cancer development?

**Findings:**

In this 60-year follow-up of a clinical trial of the BCG vaccine that included 2963 participants vaccinated at a median age of 8 years, those who received the BCG vaccine had a subsequent lung cancer rate of 18.2 cases per 100 000 person-years. Participants who received the placebo had a lung cancer rate of 45.4 cases per 100 000 person-years.

**Meaning:**

The findings suggest that receiving BCG vaccination during early childhood is associated with reduced risk of subsequent lung cancer development.

## Teaser

This 60-year follow-up of a clinical trial investigates the association between BCG vaccination during childhood and diagnosis with cancer in adulthood.

## Introduction

Live attenuated *Mycobacterium bovis* BCG vaccine was developed to prevent tuberculosis (TB), consistently a leading infectious cause of death worldwide.^1^ The BCG vaccine is currently administered to infants or children in countries with high TB prevalence and is the only vaccine approved by the World Health Organization to prevent TB. It is highly effective at preventing extrapulmonary disseminated TB and TB meningitis, but trials have shown inconsistent efficacy against pulmonary TB.^2^ In some cases, the BCG vaccine’s protective effect against TB may persist for more than 50 years.^3^ In addition to preventing TB infection, BCG is also the standard of treatment care for non–muscle invasive bladder cancer and has been associated with several immunomodulatory effects.^4,5^

Reports on BCG trials (with 13- to 27-year follow-up periods) conducted in Puerto Rico,^6^ Georgia and Alabama,^7^ and Great Britain^8^ and an observational study^9^ in New Zealand suggested that BCG vaccination was associated with increased incidence of and mortality due to leukemia and lymphoma, particularly non-Hodgkin lymphoma. Other analyses^10,11,12^ suggested no difference or decreased leukemia incidence and mortality. A systematic review^13^ showed a nonsignificant reduction in childhood leukemia (summary odds ratio, 0.73; 95% CI, 0.50-1.08) among BCG vaccine recipients. These data raised a question as to whether BCG vaccination was associated with an increased risk for subsequent lymphomas. Information about malignant neoplasm development was therefore collected during a 60-year follow-up analysis of a clinical trial of BCG vaccination in American Indian and Alaska Native (AI/AN) populations.

Cancer is a principal cause of premature death in AI/AN populations. From 1999 to 2009, the leading types of cancer among AI/AN men were prostate, lung, colorectal, kidney, and bladder; the leading types of cancer among AI/AN women were breast, lung, colorectal, uterus, and kidney.^14^ Notable cancer risk factors such as tobacco use, obesity, and reduced physical activity had higher prevalence in AI/AN populations.^15^ Because of these and other behavioral factors, lung cancer in particular has been slower to decrease among AI/AN populations than among other groups. Comparisons with the general US population from 1990 to 2009 revealed significantly higher lung cancer incidence rates and higher mortality-to-incidence ratios among AI/AN populations.^14,16^ Lung cancer is the primary cause of cancer death in AI/AN men and women combined and has one of the lowest 5-year survival rates of any cancer.^16^

We report a secondary analysis to a 60-year follow-up of a clinical trial of BCG vaccination in AI/AN schoolchildren and assess whether BCG vaccination was associated with rates of subsequent cancer diagnoses. Given the high mortality rate of cancer and the low cost of BCG vaccines, our data could have important public health implications.

## Methods

The BCG vaccine clinical trial was a multicenter, randomized by alternation, placebo-controlled, single-blind trial initiated in 1935 to 1938. Details have been published previously.^17^ The trial was conducted in AI/AN schoolchildren younger than 20 years among 9 tribes at multiple sites in the United States. Eligible participants had no signs of previous TB infection or disease as determined by chest radiography and tuberculin skin testing. A total of 3287 eligible participants were systematically stratified by school district, age, and sex, and then assigned to receive either BCG vaccine or saline placebo by alternation. Two BCG strains (strain 317 and strain 575) were prepared from live BCG culture (0.1 or 0.15 mg) and administered intradermally within 3 days (most within 12 hours) of preparation.^3^ The placebo was 0.1 mL of physiological saline solution. Participants, but not investigators, were blinded to vaccine status during the initial clinical trial.

From 1992 to 1998, a retrospective record review was conducted on vaccine trial participants.^3^ The retrospective record review study received institutional review board approval at the following locations: Walter Reed Army Medical Center, Washington, DC; Walter Reed National Military Medical Center, Bethesda, Maryland; Uniformed Services University of the Health Sciences, Bethesda, Maryland; Bloomberg School of Public Health, Johns Hopkins University, Baltimore, Maryland; Indian Health Service, Rockville, Maryland; Arizona Department of Health, Phoenix; and the SEAlaska Regional Health Corporation, Juneau. As an objective of the retrospective record review, the current analysis did not require additional approval per the policies of our institution’s research office. Informed consent (either written or oral) was obtained for interviewed study participants. Reporting of the present study followed the Consolidated Standards of Reporting Trials (CONSORT) reporting guideline.

**Figure 1.**
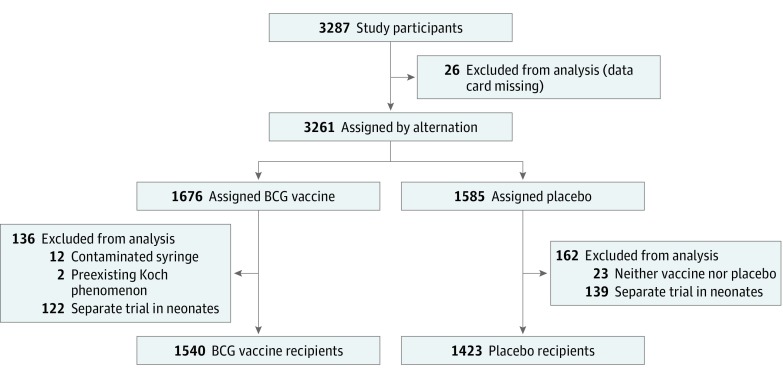
Study Flow Diagram

A total of 3287 participants were enrolled in the retrospective record review. The analysis reported in this article included 2963 participants, including 203 participants (97 in the placebo group and 106 in the BCG group) who could not be reached during the 1990s and were classified as lost to follow-up. Figure 1 shows the various exclusions made in this data set. A primary outcome, per study protocol, was to assess the risk of malignant neoplasm among the BCG and placebo groups. The association of BCG vaccination specifically with subsequent lung cancer diagnosis was not an a priori hypothesis. Cancer status was determined based on any mention of malignant tumor status, using information on death certificates, pathology reports, medical record history, or interview and was recorded using *International Classification of Diseases, Ninth Revision* codes. Only primary malignant neoplasms were included during data analysis; 10 cases of a second cancer in the cohort were excluded from the malignant neoplasm analysis. Participant medical history since the initial study (1935-1998), including tuberculosis infection (defined as a confirmed, probable, or possible case of TB), isoniazid treatment and prophylaxis, additional BCG vaccination, health risk factors, medical conditions, vital status, and other basic demographic information was systematically recorded on standardized forms from medical record review, Indian Health Service and state TB registries, death certificates, and original data cards and supplemented when needed with telephone interviews, face-to-face interviews, or mailed questionnaire responses.^3^ Causes of mortality were primarily obtained from death certificates. Investigators were blinded to participant vaccine assignment during the follow-up data collection phase.

### Statistical Analysis

Statistical analysis was completed between August 2018 and July 2019. Cox proportional hazards models were used to estimate hazard ratios (HRs) and 95% confidence intervals using R statistical software version 3.5.2 (R Project for Statistical Computing). The proportional hazards assumption was checked using Schoenfeld residuals (R function cox.zph() in the survival package) and by examining the deviance residuals and dfbeta outliers (R function ggcoxdiagnostics() in the survminer package). A competing risk analysis for lung cancer to account for death from other causes was completed using the cumincidence() function and cmprsk package.

Vaccine association with various cancers over time was examined by fitting Cox proportional hazards models from the age of participant BCG vaccination to the following in order of applicability: age at development of relevant cancer, age at death, or age at final follow-up. Independent variables included BCG vaccination, sex, region (Alaska, Arizona, North Dakota, South Dakota, and Wyoming), tribe, alcohol overuse, smoking status, vaccine strain, and prior tuberculosis infection. Cox regression models were estimated to determine the association of the indicated variable either alone (“HR”) or in combination with the vaccine variable (“Vaccine HR”) with lung cancer development. Mann-Whitney *U* tests were used to compare numerical data between treatment groups. *P* values less than .05 were considered significant using 2-tailed tests; a Bonferroni adjustment was applied for testing multiple cancer types. No power calculations for sample size determination were done as this is a follow-up of a completed trial.

## Results

A total of 3287 participants were enrolled during the original trial between 1935 to 1938; after exclusions, 1540 participants were in the BCG group and 1423 were in the placebo group. In all, 805 participants (52%) in the BCG group and 710 (50%) in the placebo group were female. The study population was similarly distributed by treatment group in terms of other demographic characteristics (Table 1). Vaccination occurred at a median (interquartile range) age of 8 (5-11) years and follow-up occurred at a median (interquartile range) age of 60 (45-65) years. At the time of follow-up, 97 participants (7%) in the placebo group and 106 participants (7%) in the BCG vaccine group could not be located; total mortality was 633 participants (44%) in the placebo group and 632 participants (41%) in the BCG group.

**Table 1.** Characteristics of Participants

Characteristic	No. (%)	
	BCG Vaccine (n = 1540)	Placebo (n = 1423)
Age at vaccination, median (IQR), y	8 (5-11)	8 (5-11)
Follow-up since vaccination, median (IQR), y	55 (37-57)	55 (36-57)
Sex		
Male	735 (48)	713 (50)
Female	805 (52)	710 (50)
Region		
Alaska	494 (32)	460 (32)
Arizona	356 (23)	348 (25)
North Dakota	208 (14)	185 (13)
South Dakota	256 (17)	241 (17)
Wyoming	226 (15)	189 (13)
Tribe		
Akimel O’odham	308 (20)	307 (22)
Arapaho	115 (7)	106 (7)
Haida	50 (3)	64 (4)
Chippewa	204 (13)	183 (13)
Shoshone	111 (7)	83 (6)
Sioux	253 (16)	238 (17)
Tlingit	360 (23)	299 (21)
Tohono O’odham	35 (2)	32 (2)
Tsimshian	59 (4)	70 (5)
Other	25 (2)	17 (1)
Unknown	20 (1)	24 (2)
BCG vaccine strain^a^		
317	1046 (68)	963 (68)
575	494 (32)	460 (32)
Smoking history		
Ever	526 (34)	460 (32)
Never	660 (43)	604 (42)
Data missing	354 (23)	359 (26)
Alcohol overuse history		
Yes	417 (27)	370 (26)
No	808 (52)	765 (54)
Data missing	315 (20)	288 (20)
Isoniazid use		
Treatment	29 (2)	58 (4)
Prophylaxis	258 (17)	197 (14)
None	936 (61)	904 (64)
Data missing	317 (21)	264 (19)
Age at isoniazid use, median (IQR), y	44 (38-48)	43 (35-50)
Tuberculosis history	151 (10)	340 (24)
Age at tuberculosis diagnosis, median (IQR), y	22 (15-40)	17 (13-27)

Abbreviation: IQR, interquartile range.

^a^ BCG vaccine strain 317 was obtained from Dr Albert Calmette via Park via King to Phipps Institute in Philadelphia, Pennsylvania (1928); strain 575 was obtained from Dr Camille Guérin from the Pasteur Institute (1938).

There were a total of 325 reported malignant neoplasms (240 per 100 000 person-years). The total cancer incidence was not significantly different between the BCG group (222 per 100 000 person-years) and the placebo group (262 per 100 000 person-years) (HR, 0.82; 95% CI, 0.66-1.02) (eTable 1 in the Supplement). There were 42 reported cases of lung cancer. Lung cancer incidence was significantly lower in the BCG vaccine group (18 cases per 100 000 person-years) than the placebo group (45 cases per 100 000 person-years) (HR, 0.39; 95% CI, 0.20-0.76); this represents an approximately 2.5-fold lower rate of lung cancer among BCG vaccine recipients (Table 2). No other types of malignant neoplasms were significantly different between the 2 trial groups; notably, leukemia and lymphoma rates were similar in the BCG group vs placebo group (HR, 0.80; 95% CI, 0.35-1.82). Ten individuals had a second malignant neoplasm, including cancers of the skin, breast, uterus, ovary, and pancreas and leukemia.

**Table 2.** Selected Cancer Rates by Type^a^

Cancer Type	BCG Vaccine, Cases per 100 000 Person-Years, No.	Placebo, Cases per 100 000 Person-Years, No.	HR (95% CI)^b^	*P* Value	Adjusted *P* Value^c^
Lung	18.2 (13)	45.4 (29)	0.39 (0.20-0.76)	.005^d^	.03^d^
Breast	29.4 (21)	37.6 (24)	0.76 (0.42-1.37)	.36	>.99
Cervix	23.8 (17)	29.8 (19)	0.80 (0.42-1.54)	.51	>.99
Colorectum	15.4 (11)	20.4 (13)	0.74 (0.33-1.65)	.46	>.99
Leukemia or lymphoma	15.4 (11)	18.8 (12)	0.80 (0.35-1.82)	.60	>.99

Abbreviation: HR, hazard ratio.

^a^ Only cancer types with 20 or more total cases were included (see eTable 1 in the Supplement for more information).

^b^ Statistical testing was performed using the Cox proportional hazards Wald test with 95% CI.

^c^ A Bonferroni adjustment for multiple testing is also provided for the indicated cancer types.

^d^ Statistically significant at *P* < .05.

Higher rates of cancer were found among female participants (280 cases per 100 000 person-years) than among male participants (197 cases per 100 000 person-years), but higher rates of lung cancer were found among men (40 cases per 100 000 person-years) than among women (23 cases per 100 000 person-years). Male BCG vaccine recipients had significantly reduced lung cancer incidence (HR, 0.35; 95% CI, 0.15-0.83), while the reduction was not statistically significant in female BCG vaccine recipients (HR, 0.49; 95% CI, 0.18-1.36). Lung cancer rates were reduced in all 5 states and all 9 tribal groups among BCG vaccine recipients, suggesting that the association of the vaccine with lung cancer was independent of geographical, cultural, or genetic differences between the populations. The Arapaho (Wyoming), Sioux (South Dakota), and Chippewa (North Dakota) had the highest risk of lung cancer, and the Akimel O’odham (Arizona) and Tohono O’odham (Arizona) peoples had the lowest risk. The lung cancer mortality rate was 13 deaths per 100 000 person-years in BCG recipients vs 41 deaths per 100 000 person-years in placebo recipients (HR, 0.32; 95% CI, 0.15-0.68) (eTable 2 in the Supplement).

Other potential risk factors for lung cancer examined in addition to sex, region, and tribe were BCG strain, smoking history, alcohol overuse, TB, and isoniazid use. The significance of the reduction in lung cancer among the BCG-vaccinated group was not affected by adjustment for any of these individual risk factors (Table 3). Isoniazid treatment and TB were both more common in the placebo group, reflecting previously published results.^3^ Although smoking was more common among male participants (529 men [36.5%] reported smoking vs 457 women [30.2%]), the combination of smoking status and sex was not associated with significance of lung cancer decrease in the BCG group (HR, 0.40; 95% CI, 0.21-0.77).

**Table 3.** Lung Cancer Cases Analyzed by Risk Factor

Risk Factor	BCG Vaccine, Cases per 100 000 Person-Years, No.	Placebo, Cases per 100 000 Person-Years, No.	HR (95% CI)^a^	*P* Value	Vaccine^b^	
					HR (95% CI)	*P* Value
Vaccine	18.2 (13)	45.4 (29)	0.39 (0.20-0.76)	.005^c^		
Sex						
Male	21.4 (7)	60.0 (19)	2.03 (1.09-3.78)	.03^c^	0.40 (0.21-0.78)	.007^c^
Female	15.6 (6)	31.1 (10)	1 [Reference]			
Region						
Alaska	17.0 (4)	42.6 (9)	3.85 (0.87-17.09)	.08	0.38 (0.20-0.74)	.004^d^
Arizona	0.0 (0)	13.0 (2)	1 [Reference]			
North Dakota	38.5 (4)	65.5 (6)	6.42 (1.41-29.30)	.02^c^		
South Dakota	36.2 (4)	68.8 (7)	7.80 (1.73-35.19)	.01^c^		
Wyoming	9.5 (1)	62.3 (5)	4.45 (0.92-22.52)	.06		
BCG vaccine strain						
317	18.8 (9)	46.8 (20)	1 [Reference]		0.39 (0.20-0.76)	.005^c^
575	17.0 (4)	42.6 (9)	0.84 (0.43-1.61)	.60		
Smoking history						
Ever	35.2 (10)	55.8 (6)	2.93 (1.32-6.54)	.01^c^	0.39 (0.20-0.75)	.005^c^
Never	6.2 (2)	20.3 (14)	1 [Reference]			
Data missing	97.4 (1)	97.4 (9)	7.57 (2.98-19.22)	<.001^c^		
Alcohol overuse history						
Yes	14.8 (3)	32.5 (6)	0.36 (0.15-0.86)	.02^c^	0.39 (0.20-0.75)	.004^c^
No	24.0 (10)	40.4 (15)	1 [Reference]			
Data missing	0.0 (0)	97.6 (8)	0.25 (0.08-0.82)	.02^c^		
Isoniazid use						
Treatment	0.0 (0)	70.4 (2)	2.65 (1.31-5.38)	.007^c^	0.37 (0.19-0.72)	.003^c^
Prophylaxis	43.8 (6)	67.5 (7)	2.55 (0.59-10.97)	.21		
None	15.1 (7)	28.1 (12)	1 [Reference]			
Data missing	0.0 (0)	101.1 (8)	5.08 (2.22-11.66)	<.001^c^		
Tuberculosis history						
Any TB	0.0 (0)	87.2 (3)	0.63 (0.23-1.77)	.38	0.37 (0.19-0.72)	.003^c^
Pulmonary TB	0.0 (0)	36.5 (2)	0.78 (0.19-3.21)	.53		
Pulmonary and extrapulmonary TB	0.0 (0)	50.7 (1)	1.25 (0.17-9.13)	.85		
No TB	19.4 (13)	47.3 (26)	1 [Reference]			

Abbreviations: HR, hazard ratio; TB, tuberculosis.

^a^ Statistical testing performed using the Cox proportional hazards Wald test with 95% CI.

^b^ Vaccine HR and Vaccine *P* value represent the significance of BCG vaccine treatment in a Cox proportional hazards Wald test with the additional variable of interest.

^c^ Statistically significant at *P* < .05.

Malignant neoplasms of the lung were classified by tissue type as small cell, non–small cell, and unknown categories, with non–small cell tumors further subdivided into adenocarcinoma, squamous cell carcinoma, and unknown categories, and compared by vaccine group (eTable 2 in the Supplement). Tumors of unspecified cell type were significantly more common in the BCG-vaccinated group. No other significant differences were found; given the large number of unspecified cell types combined with a relatively small number of lung cancer cases, this analysis was primarily exploratory.

**Figure 2.**
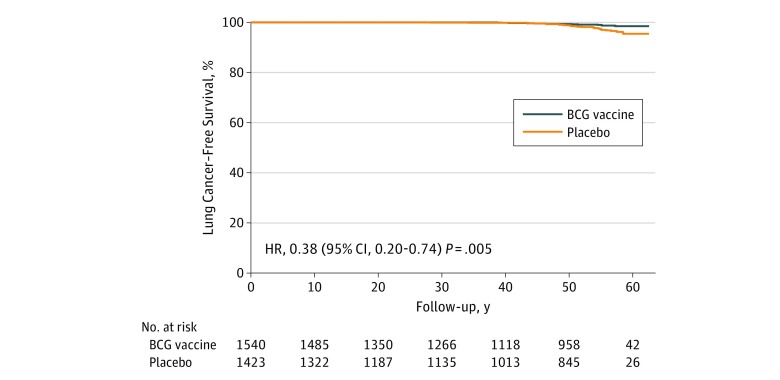
Survival Analysis for Lung Cancer in BCG Vaccine and Placebo Groups Cox regression for lung cancer development, controlling for sex, region, alcohol overuse, smoking, and tuberculosis status. All statistical testing performed using the Cox proportional hazards Wald test with 95% CI for variable significance. HR indicates hazard ratio.

Cox regression for lung cancer–free survival in the BCG-vaccinated and placebo groups controlling for sex, region, smoking, alcohol overuse, and TB revealed that BCG vaccination was significantly negatively associated with lung cancer (18.2 cases per 100 000 person-years in the BCG group vs 45.4 cases per 100 000 person-years in the placebo group; HR, 0.38; 95% CI, 0.20-0.74; *P* = .005) (Figure 2). Sex was not significant in the model (HR, 1.74; 95% CI, 0.93-3.27). Compared with living in Arizona, living in North Dakota or South Dakota was significant and positively associated with lung cancer (North Dakota HR, 4.86; 95% CI, 1.05-22.47; and South Dakota HR, 5.92; 95% CI, 1.30-27.04); living in Alaska or Wyoming showed no significant difference (Alaska HR, 3.63; 95% CI, 0.81-16.28; and Wyoming HR, 4.51; 95% CI, 0.90-22.51). Smoking history or missing smoking history were significant and positively associated with lung cancer (smoking HR, 2.44; 95% CI, 1.09-5.51; and missing smoking HR, 5.29; 95% CI, 2.04-13.67). Alcohol overuse or missing alcohol history were significant and negatively associated with lung cancer (alcohol overuse HR, 0.33; 95% CI, 0.14-0.80; and missing alcohol overuse HR, 0.26; 95% CI, 0.08-0.84). The association of alcohol overuse with lung cancer likely reflects competing risks (ie, liver failure, pancreatitis, and other cancers). Notably, prior TB was not significantly associated with lung cancer (HR, 0.66; 95% CI, 0.23-1.90).

Given the higher death rate in the placebo group, a competing risk analysis was examined that showed that the cumulative incidence curves for BCG and placebo were not significantly different for death from other causes (cumulative incidence at 60 years for BCG, 0.47 [95% CI, 0.44-0.50]; and cumulative incidence for placebo, 0.50 [95% CI, 0.47-0.54]; *P* = .12), but there remained a significant difference in lung cancer rates between treatment groups (BCG group incidence, 0.01 [95% CI, 0.01-0.02]; and placebo group incidence, 0.03 [95% CI, 0.02-0.04]; *P* = .005).

## Discussion

In this analysis, BCG vaccine recipients had a significantly lower risk of lung cancer than placebo recipients (HR, 0.38; 95% CI, 0.20-0.74) controlling for sex, region, alcohol overuse, smoking status, and TB. This represents an associated 2.5-fold decrease in lung cancer among those receiving BCG vaccine. Lung cancer was the only cancer type that was associated with BCG vaccination in this study; to our knowledge, this is the first analysis to show a protective association of BCG vaccination with lung cancer. Of note, the participants in the original trial were first vaccinated at school age (median [IQR], 8 [5-11] years), not as newborns. The effects associated with BCG vaccination appeared to persist for a long period in this cohort based on immunologic measures such as the tuberculin skin test.^18^

The results of this study are consistent with existing research indicating that BCG vaccination is associated with diverse immunological effects independently of TB. We initially anticipated that BCG vaccination might protect against lung cancer by preventing TB infection, as the granulomatous inflammatory response from TB may be favorable to malignant neoplasms known as *scar carcinomas*.^19^ Previous studies have suggested that persons infected with TB may have excess mortality from lung cancer, especially among heavy smokers.^20^ Here, BCG vaccination was associated with lower risk of lung cancer independently of TB; only 3 cases of lung cancer, all among the placebo group, were found in participants with prior TB infection. This suggests that the association is mediated directly by the immune response to BCG.

The BCG vaccination appears to be associated with beneficial nonspecific effects against various infections and disorders.^5^ A systematic review of 5 clinical trials found that BCG vaccination during infancy was associated with decreased all-cause mortality, especially from sepsis and respiratory infections, in children younger than 5 years; this association was not accounted for by reduction in rates of TB among BCG vaccine recipients.^21^ Evidence from human trials and murine models has shown that BCG vaccination was associated with lower rates of various other nonmycobacterial pathogens, including *Candida,*^22^ *Plasmodium*,^23^ an attenuated yellow fever virus,^24^ and others.^24^ The BCG vaccination is also associated with reduced hyperglycemia in patients with type 1 diabetes and may be a potential treatment.^25^ Bacille Calmette-Guérin infection in mice was associated with suppressed autoimmune responses in experimental autoimmune encephalitis^26^ and a crossover clinical trial using BCG as an adjuvant showed benefits for patients with multiple sclerosis.^27^

There are 3 current hypotheses for how BCG vaccination could mediate nonspecific effects: cross-reactive T-cell receptors and antibodies, the so-called bystander effect, and trained immunity.^28^ The first hypothesis, cross-reactive T-cell receptors and antibodies, proposes that BCG vaccination generates memory T cells and B cells that are cross-reactive with the pathogen of interest. This hypothesis is unlikely because antibodies that are cross-reactive to common antigens on BCG and pathogens the BCG vaccine protects against have yet to be found; it is also unlikely to be the mechanism for BCG protection against lung cancer for this reason. The second hypothesis suggests BCG stimulates helper T1 and helper T17 immune cell polarization and generation of memory CD4^+^ and memory NK cells; upon secondary infection with a nonmycobacterial pathogen, these memory cells undergo bystander lymphocyte activation because less stimulus is required.^29^ The third hypothesis is trained immunity, in which innate immune cells have analogous durable memory responses to unrelated secondary antigens as a result of epigenetic remodeling on primary exposure. Monocytes and NK cells are known to downregulate the inhibitory H3K9me3 and upregulate permissive H3K4me3 histones on genes of inflammatory cytokines and glycolysis regulators in response to BCG exposure.^22,30,31^ It is unknown whether reprogramming of these and other innate immune cells could lead to a heterologous response against early malignant neoplasms of the lung. Netea and colleagues^32^ have hypothesized that trained immunity may be useful for immunotherapy or vaccination against cancer.

Lung cancer was the only cancer type that decreased in association with receiving the BCG vaccination in this study. Lung adenocarcinoma and lung squamous cell carcinoma have the highest mutation frequencies of the major types of cancer; many of these mutations are in critical proteins that regulate genomic integrity, cell cycle, or MAPK signaling and have strongly immunostimulatory properties.^33^ A variety of key inflammatory factors are also released during lung cancer, including reactive oxygen species, metalloproteinases, interleukins, and interferons.^34^ These features are important in determining survival and proliferation of cancer cells, angiogenesis, metastasis, and response of the tumor to immunotherapy. The systemic changes in the immune response associated with BCG vaccination may therefore simply be more obvious for lung cancer. However, this does not explain why decreased rates of other immunologically active cancers (ie, bladder cancer, melanoma, head and neck cancer) were not found in the BCG vaccine group of this study. It is possible that the BCG association might be broader (ie, associations with other types of cancer) but masked in this report owing to small numbers of cases. Alternatively, BCG vaccination may have more significant effects on immunoregulation in the lung environment. Intradermal administration of BCG vaccine in mice was associated with increased recruitment of innate lymphoid cells and NK cells to the lung.^35^ Another murine study implicated airway-resident memory T cells in BCG-related immunity.^36^ Overall, more complete analyses of the lung’s response to BCG vaccination need to be conducted in human trials.

Treatment with BCG in previous lung cancer clinical trials has been met with limited to no success.^37,38^ The immunity of prevention and treatment are different; BCG might function as an effective preventive measure but not as a treatment for lung cancer. The BCG vaccine was not associated with a decreased rate of bladder cancer in this study (HR, 1.34; 95% CI, 0.22-8.03).

### Strengths and Limitations

This study has some strengths. Our study can make conclusions about the association of BCG vaccine and reduced lung cancer with data acquired from a clinical trial. There is likely greater external validity of our findings because the study was conducted on multiple genetically distinct subpopulations with likely different cancer rates, and participants were followed up for long enough to reach an age of increased cancer risk. Additionally, we found similar results for the 2 BCG vaccine strains, which may have different viability, genetics, and immunogenicity.

However, this study also has limitations. Aside from randomization by alternation, we were unable to control for other confounding factors for lung cancer, such as obesity, wood fire cooking and environmental pollution, physical inactivity, radon, radiation exposure, secondhand smoke, or asbestos. In addition, this study is subject to the limitations of a post hoc analysis. Missing or unspecified data were present for some variables, but we have no reason to believe that this differentially affected the randomized subgroups.

## Conclusions

Long-term follow-up of an AI/AN population immunized with the BCG vaccine at an early age shows that lung cancer was significantly less common in those receiving BCG vaccine vs placebo, with no other cancer rates significantly different by vaccine group. While BCG vaccine was associated with preventing TB in this clinical trial with long-term protective effects, the observed decrease in lung cancer was not associated with prior TB infection. The mechanism of this observed protection is unknown, but the association is large and scientifically plausible; we favor trained immunity as a hypothesis. This finding should be further corroborated and evaluated in other long-term BCG study cohorts such as in the Medical Research Council BCG trial in the United Kingdom and the BCG vaccination program in Norway.

## Notes

Supplement. **eTable 1.** Additional Malignancy Rates by Body Site **eTable 2.** Various Outcomes for Participants With Lung Malignancies
