# Supplementary material for: Association of BCG Vaccination in Childhood With Subsequent Cancer Diagnoses: A 60-Year Follow-up of a Clinical Trial
Source: JAMA Netw Open. 2019 Sep 25;2(9):e1912014. doi: 10.1001/jamanetworkopen.2019.12014 (PMC6763973; doi:10.1001/jamanetworkopen.2019.12014)
Supplement: Supplement. — eTable 1. Additional Malignancy Rates by Body Site eTable 2. Various Outcomes for Participants With Lung Malignancies [file jamanetwopen-2-e1912014-s001.pdf]

Supplementary Online Content

Usher NT, Chang S, Howard RS, et al. Association of BCG vaccination in childhood with subsequent cancer diagnoses: a 60-year follow-up of a clinical trial. *JAMA Netw Open*. 2019;2(9):e1912014. doi:10.1001/jamanetworkopen.2019.12014

eTable 1. Additional Malignancy Rates by Body Site

eTable 2. Various Outcomes for Participants with Lung Malignancies

This supplementary material has been provided by the authors to give readers additional information about their work.

© 2019 Usher NT et al. *JAMA Network Open.*

eTable 1. Additional malignancy rates by body site.

Cancer type	BCG	Placebo	HR (95% CI)	P-value
Lung	18.2 (13)	45.4 (29)	0.39 (0.20, 0.76)	0.005*
Breast	29.4 (21)	37.6 (24)	0.76 (0.42, 1.37)	0.36
Cervix	23.8 (17)	29.8 (19)	0.80 (0.42, 1.54)	0.51
Colorectum	15.4 (11)	20.4 (13)	0.74 (0.33, 1.65)	0.46
Leukemia/lymphoma	15.4 (11)	18.8 (12)	0.80 (0.35, 1.82)	0.60
Prostate	12.6 (9)	14.1 (9)	0.85 (0.34, 2.15)	0.74
Skin	4.2 (3)	11.0 (7)	0.36 (0.09, 1.41)	0.14
Uterus	15.4 (11)	9.4 (6)	1.63 (0.60, 4.40)	0.34
Gallbladder	7.0 (5)	9.4 (6)	0.72 (0.22, 2.35)	0.58
Head and neck	12.6 (9)	7.8 (5)	1.60 (0.54, 4.76)	0.40
Ovary	7.0 (5)	7.8 (5)	0.89 (0.26, 3.07)	0.85
Stomach	4.2 (3)	7.8 (5)	0.53 (0.13, 2.24)	0.39
Kidney	12.6 (9)	4.7 (3)	2.63 (0.71, 9.70)	0.15
Esophagus	2.8 (2)	4.7 (3)	0.57 (0.10, 3.44)	0.54
Bladder	4.2 (3)	3.1 (2)	1.34 (0.22, 8.03)	0.75
Brain	2.8 (2)	3.1 (2)	0.88 (0.12, 6.26)	0.90
Thyroid	2.8 (2)	3.1 (2)	0.88 (0.12, 6.24)	0.90
Liver	1.4 (1)	3.1 (2)	0.47 (0.04, 5.16)	0.53
Pancreas	7.0 (5)	1.6 (1)	4.47 (0.52, 38.27)	0.17
Melanoma	1.4 (1)	1.6 (1)	0.89 (0.06, 14.19)	0.93
Other	5.6 (4)	4.7 (3)	1.18 (0.26, 5.27)	0.83
Unspecified	15.4 (11)	12.5 (8)	1.17 (0.47, 2.91)	0.74
Total	221.5 (158)	261.6 (167)	0.82 (0.66, 1.02)	0.07

Data are cases per 100,000 person-years (n). Statistical testing performed using the Cox proportional hazards Wald test with 95% CI. A total of n=325 cases of cancer were reported. *p<0.05.

© 2019 Usher NT et al. *JAMA Network Open.*

eTable 2. Various outcomes for participants with lung malignancies.

			BCG	Placebo	HR (95% CI)	P-value
Malignancy cell type	Non-small cell	Total	9.8 (7)	14.1 (9)	0.69 (0.26, 1.84)	0.46
		Adenocarcinoma	4.2 (3)	0.0 (0)		
		Squamous cell	4.2 (3)	4.7 (3)		
		Unspecified	1.4 (1)	9.4 (6)		
	Small cell		1.4 (1)	9.4 (6)	0.15 (0.02, 1.24)	0.08
	Unspecified		7.0 (5)	21.9 (14)	0.31 (0.11, 0.86)	0.02*
Lung cancer mortality			12.6 (9)	40.7 (26)	0.32 (0.15, 0.68)	0.003*
Age of diagnosis (years)			59 (52-62)	59 (56-64)		0.65
Age of death (years)			60 (57-62)	60 (57-65)		0.59

Data are cases per 100,000 person-years (n) or median (IQR). Statistical testing performed using the Cox proportional hazards Wald test with 95% CI. For age outcomes, groups compared by Mann-Whitney U test. *$p<0.05$.

© 2019 Usher NT et al. *JAMA Network Open.*
